# Shanxi Aged Vinegar Protects against Alcohol-Induced Liver Injury via Activating Nrf2-Mediated Antioxidant and Inhibiting TLR4-Induced Inflammatory Response

**DOI:** 10.3390/nu10070805

**Published:** 2018-06-22

**Authors:** Ting Xia, Jin Zhang, Jiahui Yao, Bo Zhang, Wenhui Duan, Chaoya Zhao, Peng Du, Jia Song, Yu Zheng, Min Wang

**Affiliations:** State Key Laboratory of Food Nutrition and Safety, Key Laboratory of Industrial Fermentation Microbiology, Ministry of Education, Tianjin Engineering Research Center of Microbial Metabolism and Fermentation Process Control, College of Biotechnology, Tianjin University of Science & Technology, Tianjin 300457, China; xiatingsyu@foxmail.com (T.X.); ZHANGJIN1220@foxmail.com (J.Z.); yaojiahui1015@foxmail.com (J.Y.); bozhango@163.com (B.Z.); duanwhyy@163.com (W.D.); zcysuccess@foxmail.com (C.Z.); dp9298@163.com (P.D.); tjsongjia@tust.edu.cn (J.S.); yuzheng@tust.edu.cn (Y.Z.)

**Keywords:** alcohol-induced liver injury, Shanxi aged vinegar, oxidative stress, Nrf2, TLR4

## Abstract

Shanxi aged vinegar (SAV) is a typical fermented and antioxidant food, which has various health-promoting effects. This work aimed to explore the effects of SAV on alcohol-induced liver injury. A mice model of alcoholic liver injury was established to illuminate its potential mechanisms. All mice pretreated with SAV and then received an ethanol solution (50% *w*/*v*, 4.8 g/kg b.w.). The results showed that SAV ameliorated alcohol-induced histological changes and elevation of liver enzymes. SAV attenuated alcohol-induced oxidative stress by declining levels of hepatic oxidants, and restoring depletion of antioxidant enzyme activities in mice livers. Moreover, SAV alleviated alcohol-induced oxidative damage by activating nuclear factor erythroid-2-related factor 2 (Nrf2)-mediated signal pathway. In addition, SAV prevented alcohol-induced inflammation by suppressing lipopolysaccharide (LPS) level and activities of pro-inflammatory enzymes, and regulating inflammatory cytokines. SAV inhibited alcohol-induced inflammation through down-regulating the expression of Toll-like receptor 4 (TLR4)-mediated inflammatory response. The findings provide crucial evidence for elucidating the hepatoprotective mechanisms of SAV and encourage the future application of SAV as a functional food for liver protection.

## 1. Introduction

Alcohol is primarily metabolized in liver, and excess alcohol consumption can induce liver damage [1]. It has been demonstrated that alcohol-induced liver injury is one of the leading causes of alcohol-related death, which is characterized by progressive damage of the liver from fibrosis to cirrhosis [2,3]. Acute alcohol consumption leads to elevation of liver function enzymes, acute liver inflammation and metabolism disorders including hyperlactacidemia, hyperuricemia, enhanced lipogenesis and depressed lipid oxidation [4,5]. However, chronic alcohol exposure induces continuous and progressive inflammation and lipid metabolism disorders, which cause alcoholic hepatitis, fibrosis, cirrhosis and even hepatocarcinoma [6].

Several studies have revealed that oxidative stress and inflammation play important roles in the pathogenesis of acute or chronic alcohol-induced liver injury [7,8]. Alcohol consumption can cause over production of reaction oxygen species (ROS). On the one hand, excess ROS induces the depletion of enzymatic and non-enzymatic antioxidants, resulting in oxidative stress in the liver [9]. On the other hand, excess ROS also induces formation of lipid radicals and accumulation of lipid in hepatocytes, which lead to lipid peroxidation [10]. Additionally, alcohol accumulation destroys the integrity of hepatocyte membrane and promotes the release of hepatic enzymes, such as alanine aminotransferase (ALT) and aspartate aminotransferase (AST) [11]. Alcohol consumption also disrupts intestinal barrier functions and causes bacterial endotoxin (lipopolysaccharide, LPS) to permeate into the systemic circulation, which induces inflammation [12]. Toll-like receptor 4 (TLR4), a receptor of LPS in Kupffer cells, induces the activation of the nuclear factor-kappa B (NF-κB). It can up-regulate the expressions of pro-inflammatory enzymes, such as inducible nitric oxide synthase (iNOS) and cyclooxygenase-2 (COX-2), subsequently increase the production of pro-inflammatory cytokines [13,14]. Therefore, preventing oxidative stress and inflammation would be effective in delaying the pathogenesis of alcoholic liver injury.

It has been reported that many natural substances are rich in antioxidant ingredients and exhibit high antioxidant capability [15]. These natural antioxidants are beneficial to human body because they can scavenge free radicals and act as reductants [16,17,18]. Arteel et al. found that green tea contained antioxidant polyphenoly and exhibited hepatoprotective effects against alcohol-induced liver injury in vivo [19]. Vinegars are natural and fermented foods, which have antioxidant ingredients [20]. Their antioxidant capability is associated with phenols, flavonoids and melanoidins produced by its raw materials and microbiological actions [21]. Several studies have demonstrated that vinegars possess a variety of physiological functions, including anti-oxidation, anti-inflammation and anti-obesity [22,23]. Chou et al. reported that black vinegar exhibited lipid-lowering and antioxidant effects on high-fat and cholesterol-diet fed hamsters [24]. Shanxi aged vinegar, as a traditional Chinese vinegar, is produced by solid-state fermentation techniques [25]. Our previous study demonstrated that SAV exhibited high antioxidant capacity and protected hepatocytes against oxidative damage in vitro [26]. However, the effects of SAV against alcohol-induced liver injury in vivo remain unclear.

The aim of our present study was to explore the possible protective effects of SAV on acute alcoholic liver injury by detecting oxidative stress indicators and inflammatory markers in mice. Then, the potential molecular mechanisms of SAV were investigated by examining antioxidant and inflammatory signal pathways. These findings would elucidate the underlying protective mechanisms of SAV on alcohol-induced acute liver injury.

## 2. Materials and Methods

### 2.1. Chemicals and Reagents

Acetic acid and absolute ethanol (≥99.8%) were purchased from Sigma-Aldrich (St Louis, MO, USA). The enzyme-linked immunosorbent assay (ELISA) kits were purchased from eBioscience (San Diego, CA, USA). The primary antibodies against rabbit nuclear factor erythroid-2-related factor 2 (Nrf2), nicotinamide quinone oxidoreductase 1 (NQO1), heme oxygenase-1 (HO-1), glutamate-cysteine ligase modifier subunit (GCLM), Phospho-IκB (p-IκB) and GAPDH were obtained from Abcam (Cambridge, MA, USA). The primary antibodies against rabbit iNOS, TLR4, myeloid differentiation factor 88 (MyD88), Phospho-NF-κB p65 (p-NF-κB p65) and the secondary horseradish peroxidase (HRP)-labelled goat-anti-rabbit and goat-anti-mouse antibodies were purchased from Cell Signaling Technology (Danvers, MA, USA). The primary antibodies against mouse COX-2 was obtained from Santa Cruz Biotechnology (Santa Cruz, CA, USA).

Shanxi aged vinegar (SAV) samples with 96 months of aging time were collected from local supermarkets. Total acid of SAV was 8.0 g/100 mL, and contents of proteins, crude fats and carbohydrates were 2.96 ± 0.20 g/100 mL, 0.90 ± 0.16 g/100 mL and 6.00 ± 0.88 g/100 mL, respectively. Acetic acid is the major composition of SAV, and the content of was 6.2 × 104 ± 1.8 × 103 μg/mL. In addition, the antioxidant components including phenols, flavonoids and melanoidins were reported in our previous study [26]. The contents of phenols and flavonoids in SAV were 2.883 ± 0.064 mg GAE/mL and 3.008 ± 0.063 mg RE/mL, respectively. The browning index (A420 nm) of melanoidins was 1.236 ± 0.029 in SAV.

### 2.2. Animals and Treatments

Experimental animals were purchased from Beijing Vital River Laboratory Animal Technology Co., Ltd. (Beijing, China). The animal experiments were executed according to the guidelines of the institutional animal ethics committee, and supported by the institutional animal committee of Nankai University. Male ICR (6–8 weeks old) rats were housed five per cage. Seventy rats were randomly assigned to seven groups, i.e., control group, ethanol group, alcohol + SAV (0.625, 1.250 and 2.500 mL/kg b.w., 14 days) groups, alcohol + SAV (2.500 mL/kg b.w., 1 day) group, and acetic acid (AA) group (2.500 mL/kg b.w.). Control and ethanol group were only treated with distilled water for 14 days. Three SAV groups were subjected to oral treatments once daily for 14 days by oral gavage, and AA group were treated with synthetic vinegar (2.500 mL/kg b.w.). At day 14, alcohol + SAV (2.500 mL/kg b.w., 1 day) group was treated with SAV (2.500 mL/kg b.w.) by oral gavage. Except for those in normal control group, mice were given by gavage with an ethanol solution (50% *w*/*v*, 4.8 g/kg b.w.) 2 h after final administration. All the mice were euthanized after 12 h, blood and liver tissues were harvested and stored at −80 °C. 

### 2.3. Histopathological Analysis

Fixed liver tissues were embedded in paraffin wax and cut in 5-μm-thick pieces. Paraffin portions were stained with hematoxylin-eosin (H & E) for histopathological examination according to a standard protocol. It has been reported that level of liver steatosis was graded as 0 point for no hepatocytes affected, 0.5 point for slight (0–5%), 1 point for mild (5–20%), 2 points for moderate (20–50%), and 3 points for severe (>50%) [27,28]. Oil Red O staining was used to determine hepatic fat visualization. Briefly, liver sections were embedded in a frozen tissue matrix, and then cut into 8 μm thin sections and stained with Oil Red O (Sigma-Aldrich) [29]. The stained sections were observed by light microscopy (Nikon, Tokyo, Japan).

### 2.4. Serum Biochemical Analysis

ALT, AST, and triglyceride (TG) assay kits were obtained from Nanjing Jiancheng Bioengineering Institute (Nanjing, China). The activities of serum ALT, AST, and TG levels in serum and liver were measured according to the commercial assay kits.

### 2.5. Determination of Hepatic ROS, MDA and Antioxidant Enzymes

Malonaldehyde (MDA), glutathione peroxidase (GSH-Px), superoxide dismutase (SOD) and catalase (CAT) assay kits were purchased from Nanjing Jiancheng Bioengineering Institute (Nanjing, China). Hepatic ROS was quantified using an ELISA kit. Hepatic level of MDA and activities of GSH-Px, SOD and CAT were measured by following the commercial kits’ protocols.

### 2.6. Measurement of Hepatic Inflammation Biomakers

iNOS and nitric oxide (NO) assay kits were obtained from Nanjing Jiancheng Bioengineering Institute (Nanjing, China). The levels of iNOS and NO in liver tissues were measured using commercial assay kits. The levels of LPS, COX-2, interleukin-1β (IL-1β), and interleukin-10 (IL-10) were quantified using ELISA kits. Optical density was measured at 450 nm with a Bio-Rad 550 microplate reader (Bio-Rad Laboratories Inc., Hercules, CA, USA).

### 2.7. Western Blot Analysis

Total proteins in liver cells were extracted using RIPA buffer with 1% phenylmethanesulfonyl fluoride (PMSF), and protein concentration was detected using a BCA protein assay kit. Equal amounts of protein (60 µg) were diluted in SDS-sample buffer and were separated by 12% sodium dodecyl sulphate–polyacrylamide (SDS-PAGE) gel. After electrophoresis, the proteins were subsequently blotted onto a polyvinylidene fluoride (PVDF) membrane (Millipore, Billerica, MA, USA). Then, the membranes were blocked with 5% non-fat milk in TBST buffer for 1 h at room temperature, and then incubated with primary antibodies specific for Nrf2, NQO1, HO-1, GCLM, TLR4, MyD88, p-IκB α, p-NF-κB p65, (Abcam Ltd., Cambridge, UK) at 4 °C for overnight. After washing with TBST for three times, the membranes were incubated with HRP-conjugated secondary antibodies for 1 h at room temperature and the immunoreactive bands were detected using an enhanced chemiluminescence (ECL, Thermo Scientific, Vantaa, Finland) [26]. Each membrane was stripped and re-probed with GAPDH antibody (Bioworld Tech., Nanjing, China) to ensure equal protein loading.

### 2.8. Statistical Analysis

All results were presented as means ± standard error of the mean (SEM). Comparisons among all groups were performed with one-way analysis of variance (ANOVA) test. Significant differences were calculated according to the Bonferroni post hoc test. A difference was considered to be statistically significant at the level of *p* < 0.05.

## 3. Results

### 3.1. Effect of SAV on Alcohol-Induced Liver Injury

**Figure 1:**
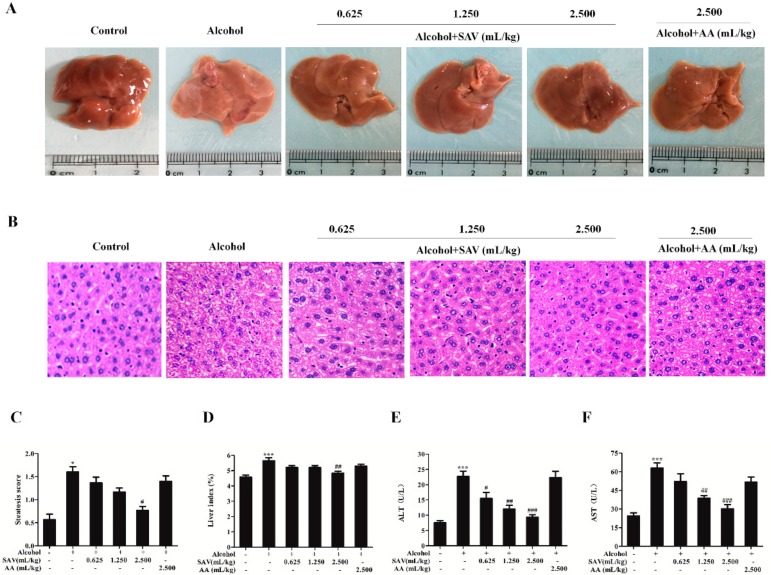
Effect of SAV on alcohol-induced liver injury. (**A**) Gross examination of mice livers; (**B**) Histological examination of liver sections stained with H&E. (200× magnification); (**C**) Liver stestosis score; (**D**) the level of liver index. Serum levels of ALT (**E**) and AST (**F**) were measured with microplate reader. All data are expressed as mean ± SEM (*n* = 5–7). * *p* < 0.05, *** *p* < 0.001 versus control group, # *p* < 0.05, ## *p* < 0.01, ### *p* < 0.001 versus ethanol group.

To evaluate protective effects of SAV, hepatic histopathological changes were evaluated by histopathological observation and H&E staining. As shown in Figure 1A, gross examination revealed that liver enlargement, macrovesicular steatosis and surface rough displayed in alcohol-treated group compared with the control group. However, these changes were alleviated by SAV pretreatment. In addition, the liver tissues from alcohol-treated mice showed vacuolar degeneration of hepatic cells and obviously swollen hepatocytes along with inflammatory cell infiltration compared with the control group. However, these pathological changes in alcohol-treated liver tissue were attenuated by SAV pretreatment. No obvious histopathological differences were observed between the AA group and the alcohol-treated group (Figure 1B). Furthermore, liver index was remarkably increased in alcohol-treated group compared with the control group, whereas that was significantly reduced by SAV pretreatment group at a dose of 2.500 mL/kg b.w. compared with the ethanol group (Figure 1D). As shown in Figure 1E,F, the levels of serum ALT and AST were dramatically increased in alcohol-treated group compared with the control group. However, these elevations were significantly reversed by SAV pretreatment at a dose of 1.250 mL/kg b.w. and 2.500 mL/kg b.w. There were no significant differences in serum ALT and AST levels between the AA group and the ethanol group. 

To further determine protective effects of SAV, a single-dose and 14-day SAV pre-treatment were compared in alcohol-treated mice. The results showed that no obvious differences in macroscopic morphology and histopathological examination were observed between the single-dose SAV group and the ethanol group (Figure S1A,B). In addition, acute ethanol exposure obviously increased the levels of ALT and AST, and this elevation was significantly decreased by 14-day SAV pre-treatment (2.500 mL/kg b.w). However, the levels of ALT and AST were slightly reduced in the single-dose SAV group (2.500 mL/kg b.w) compared with those in the ethanol group (Figure S1C,D). It is 14-day SAV pre-treatment rather than a single-dose SAV pre-treatment that can alleviate alcohol-induced liver injury in mice. Collectively, these results indicate that SAV effectively alleviates alcohol-induced liver injury in mice.

### 3.2. Effect of SAV on Alcohol-Induced Hepatic Steatosis

**Figure 2:**
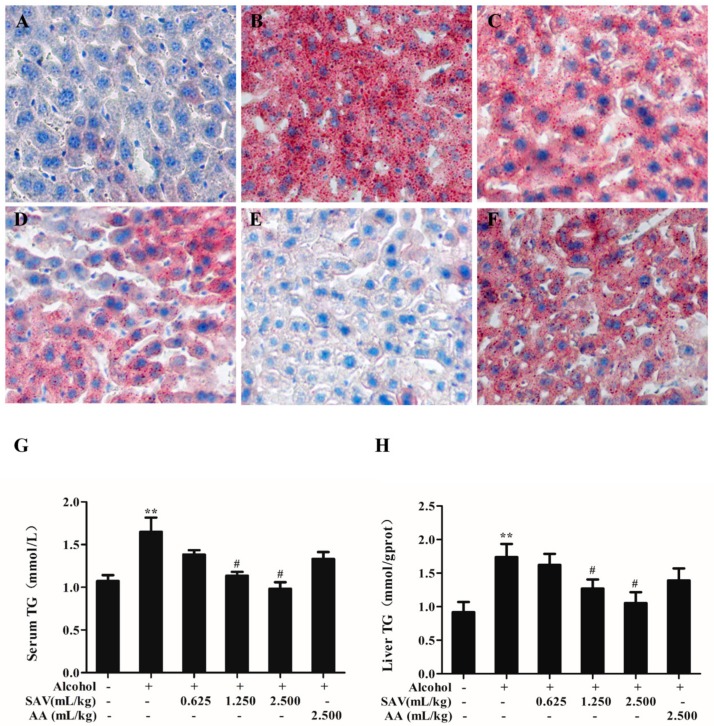
Effect of SAV on alcohol-induced hepatic steatosis. Liver sections were stained with Oil Red O and examined by light microscopy (200× magnification) in Control group (**A**); Ethanol group (**B**); Ethanol + SAV (0.625 mL/kg b.w.) group (**C**); Ethanol + SAV (1.250 mL/kg b.w.) group (**D**); Ethanol + SAV (2.500 mL/kg b.w.) group (**E**) and Ethanol + AA group (**F**); Serum level of TG (**G**) and hepatic level of TG (**H**) were detected with microplate reader. All data are expressed as mean ± SEM (*n* = 5–7). ** *p* < 0.01 versus control group versus control group, # *p* < 0.05 versus ethanol group.

To assess the effect of SAV on hepatic steatosis induced by acute ethanol exposure, lipid accumulation and lipid parameters were examined. As shown in Figure 2A,B, ethanol remarkably promoted the accumulation of lipid droplet in alcohol-treated mice livers. However, the SAV pretreatment obviously inhibited the alcohol-induced accumulation of lipid droplet (Figure 2C–E). Hepatic steatosis in the AA group was not obviously restored compared with the alcohol-treated group (Figure 2F). Meanwhile, the result showed that the level of hepatic TG in SAV-pretreated mice (1.250 mL/kg b.w. and 2.500 mL/kg b.w.) was significantly declined compared with that in alcohol-treated mice (Figure 2H). Quantitative TG determination in livers was consistent with histopathological examination. In addition, serum TG level in SAV-pretreated mice (1.250 mL/kg b.w. and 2.500 mL/kg b.w.) was significantly declined compared with that in alcohol-treated mice (Figure 2G).

Quantification analysis for steatosis was used to further evaluate cellular damage in the liver. As shown in Figure 1C, SAV pretreatment alleviated alcohol-induced hepatic steatosis, which was assessed by steatosis score. Collectively, the steatosis grade, oil red O staining and TG levels indicate that SAV can reduce alcohol-induced hepatic steatosis.

### 3.3. Effect of SAV on Alcohol-Induced Oxidative Stress

It is well known that excess alcohol consumption leads to hepatic oxidative stress, which is characterized by an imbalance between oxidants and antioxidant systems [30]. Furthermore, hepatic oxidative stress can promote the development of alcohol-induced liver injury [31]. In order to evaluate the effect of SAV on alcohol-induced oxidative stress, ROS level, lipid peroxidation and antioxidant enzyme activities were examined. As shown in Table 1, hepatic levels of ROS and MDA in alcohol-treated group were significantly higher than those in the control group. However, these elevated levels were significantly reduced by SAV pretreatment at a dose of 1.250 mL/kg b.w.and 2.500 mL/kg b.w. In addition, the activities of GSH-Px, SOD and CAT were significantly decreased in the alcohol treatment group. However, the decrease of these enzymes were restored by SAV pretreatment in a dose-dependent manner, especially at a dose of 2.500 mL/kg b.w. (*p* < 0.01). There were no significant differences in levels of ROS and MDA and activities of antioxidant enzymes between the AA group and the alcohol-treated group in mice livers. These results suggest that SAV can attenuate alcohol-induced oxidative stress via reducing ROS and MDA production and improving antioxidant capacity.

### 3.4. Effect of SAV on Nrf2-Mediated Antioxidant Response

**Figure 3:**
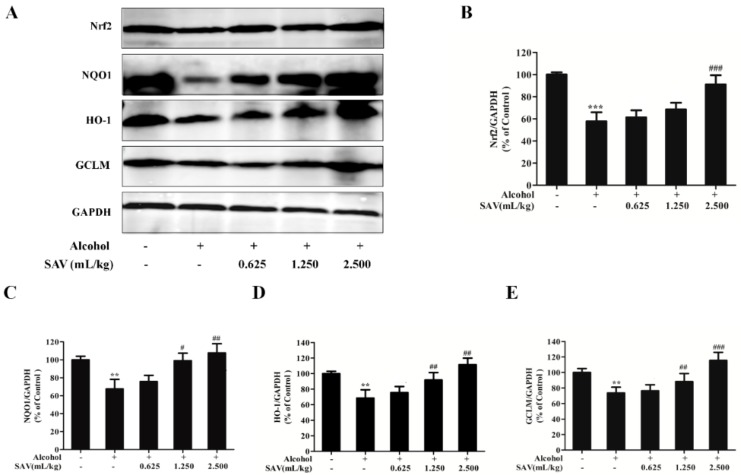
Effect of SAV on the Nrf2-mediated antioxidant response. (**A**) The expression levels of Nrf2, NQO1, HO-1 and GCLM in livers were measured by western blot analysis; (**B**) Quantification of Nrf2 expression; (**C**) Quantification of NQO1 expression; (**D**) Quantification of HO-1 expression; (**E**) Quantification of GCLM expression. All data are expressed as mean ± SEM (*n* = 5–7). ** *p* < 0.01 versus control group, *** *p* < 0.001 versus control group, # *p* < 0.05, ## *p* < 0.01, ### *p* < 0.001 versus ethanol group.

To further explore the antioxidant mechanism underlying the protective effects of SAV on alcohol-induced oxidative damage, the expressions of Nrf2 and its downstream proteins were analyzed by western blot analysis. Nrf2, a transcription factor, is essential for encoding antioxidant proteins in response to oxidative stress [32]. It promotes the transcription of several anti-oxidative genes, including HO-1, NQO1 and GCLM [33]. As shown in Figure 3A,B, alcohol exposure remarkably down-regulated Nrf2 expression, which was restored by pretreated with SAV. Then, the expressions of its downstream proteins NQO1, HO-1 and GCLM were examined. As shown in Figure 3A,C–E, the expressions of NQO1, HO-1 and GCLM in alcohol-treated group were decreased compared with the control group. However, SAV pretreatment significantly restored the expressions of these proteins compared with the alcohol-treated group. In general, these results suggest that the hepatoprotective effect of SAV against alcohol-induced oxidative damage is associated with activation of the Nrf2-mediated antioxidant response.

### 3.5. Effect of SAV on Alcohol-Induced Inflammation

**Figure 4:**
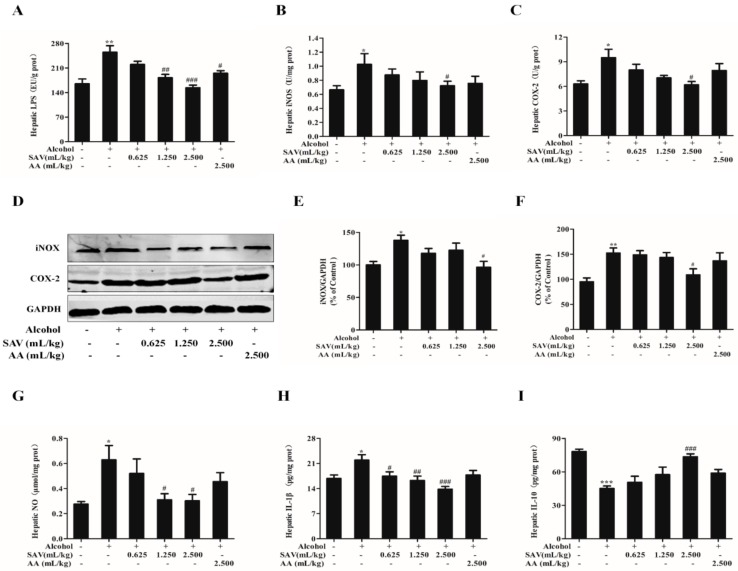
Effect of SAV on alcohol-induced inflammation. The level of LPS (**A**); the activities of iNOX (**B**) and COX-2 (**C**); (**D**) The expression levels of iNOX and COX-2; (**E**) Quantification of iNOX expression; (**F**) Quantification of COX-2 expression; (**G**) hepatic level of NO. the production of IL-1β (**H**) and IL-10 (**I**) were determined with microplate reader. All data are expressed as mean ± SEM (n = 5–7). * *p* < 0.05 versus control group, ** *p* < 0.01 versus control group, *** *p* < 0.001 versus control group, # *p* < 0.05, ## *p* < 0.01, ### *p* < 0.001 versus ethanol group.

It has been reported that excess ethanol induces the elevation of LPS, which can mediate the expressions of inflammatory factors [34]. In order to evaluate the effect of SAV on alcohol-induced inflammation, the expressions of inflammation-related factors were detected. As shown in Figure 4A, the level of LPS was remarkably increased after alcohol challenge, whereas that was significantly reduced by SAV pretreatment at a dose of 1.250 mL/kg b.w. and 2.500 mL/kg b.w. In addition, iNOS and COX-2 are key pro-inflammatory enzymes in activating pro-inflammatory cytokines production, which play important roles in ethanol-induced liver damage [35]. The results showed that the activities of iNOS and COX-2 as well as NO production were remarkably increased in the alcohol-treated group. However, these elevations were significantly restored by SAV pretreatment at a dose of 2.500 mL/kg b.w. compared with the ethanol group (Figure 4B,C,G). Meanwhile, the expressions of iNOS and COX-2 were measured by western blot. The results showed that the expressions of iNOS and COX-2 were up-regulated after alcohol exposure compared with the vehicle control, whereas these increased proteins were significantly suppressed by SAV pretreatment at a dose of 2.500 mL/kg b.w. (Figure 4D–F). The results in immunoblots were in line with these two key mediators detected by ELISA.

Furthermore, the levels of pro-inflammatory cytokine IL-1β and anti-inflammatory cytokine IL-10 were detected. SAV pretreatment significantly inhibited the increase of IL-1β and restored the decrease of IL-10 in alcohol-treated mice at a dose of 2.500 mL/kg b.w. (Figure 4E,F). There was no significant difference in the expressions of inflammatory-related factors between the AA group and alcohol-treated group. Taken together, these data demonstrate that SAV can alleviate alcohol-induced inflammation in mice.

### 3.6. Effect of SAV on TLR4-Induced Inflammatory Response

**Figure 5:**
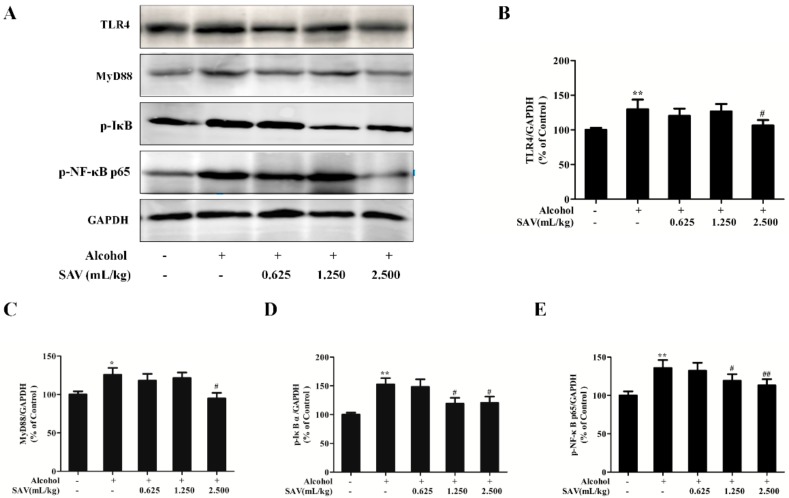
Effect of SAV on TLR4-induced inflammatory response. (**A**) The expression levels of TLR4, MyD88, p-IκBα and p-NF-κB p65 were analyzed by western blot; (**B**) Quantification of TLR4 expression; (**C**) Quantification of MyD88 expression; (**D**) Quantification of p-IκBα expression; (**E**) Quantification of p-NF-κB p65 expression. All data are expressed as mean ± SEM (*n* = 5–7). * *p* < 0.05 versus control group, ** *p* < 0.01 versus control group, # *p* < 0.05, ## *p* < 0.01 versus ethanol group.

To explore the anti-inflammatory mechanisms underlying the protective effects of SAV on alcohol-induced liver injury, the expressions of protein related to TLR4-induced signal pathway were analyzed by western blot. It is well known that TLR4, a receptor for LPS, would be activated by alcohol consumption [36]. As shown in Figure 5A,B, TLR4 expression was significantly up-regulated after alcohol exposure compared with the vehicle control. However, this elevation was suppressed by SAV pretreatment. MyD88, a major adaptor protein of TLR4, initiates the downstream signal cascade, inducing the activation of inflammatory responses [37]. As shown in Figure 5A,C–E, the expressions of MyD88, p-IκB α and p-NF-κB p65 were significantly up-regulated after ethanol exposure compared with the control group. However, the up-regulated proteins were inhibited by SAV pretreatment. Collectively, the results imply that SAV alleviates alcohol-induced liver inflammation via inhibition of TLR4-induced signal pathway.

## 4. Discussion

Excess alcohol consumption induces liver injury, which is a major cause of morbidity and mortality worldwide [38]. Oxidative stress and inflammation are two essential elements in the development and progression of alcoholic liver injury [39]. Natural antioxidants have been known as effective agents for the prevention of alcohol-induced liver injury [29]. Vinegars, as a fermented and healthy food, produce a large number of nutrients and functional factors such as organic acids, amino acids, phenols and flavonoids. Acetic acid is the main component of vinegar, which contributes to the unique flavor and aroma of vinegars [40]. Several studies have found that acetic acid exhibits antibacterial capability and reduces blood pressure and glucose levels [41,42,43]. Vinegars are also rich in antioxidant components. It has been reported that traditional balsamic vinegar (TBV) exhibited high antioxidant activity attributed to the presence of phenols and flavonoids [44]. In our previous study, antioxidant activities of SAV were highly correlated with the contents of phenolic compounds, which were detected by HPLC analysis, mainly including gallic acid, catechins and caffeic acid [26]. However, the antioxidant effect protective mechanisms of SAV against alcohol-induced liver injury in vivo have rarely been explored. In the present study, the effects of SAV on acute alcoholic liver injury and its potential mechanisms were explored in mice.

It has been reported that excess alcohol consumption leads to histopathological changes and release of hepatic enzymes [28]. The increased activities of ALT and AST in circulation reflect liver cell damage and leakage [45]. In the current study, acute alcohol consumption caused obvious pathological changes and the increase of serum ALT and AST levels. However, SAV could effectively inhibit the increase of serum ALT and AST levels in alcohol-treated mice. Meanwhile, excess alcohol consumption also causes hepatic steatosis, which is the early appearance of liver injury [46,47]. In the initial stage, alcohol consumption activates dehydrogenase and generates the reduced form of nicotinamide adenine dinucleotide. Consequently, lipid synthesis and degradation lose balance, which promotes hepatic steatosis and leads to liver injury [48]. In this study, SAV pretreatment remarkably reduced the accumulation of small lipid droplets and the elevations of serum and hepatic TG levels in the alcohol-treated group, suggesting that SAV can reduce alcohol-induced hepatic steatosis. Otherwise, there were no significant differences in liver enzymes and TG levels between the AA group and the ethanol group. Cao, et al., found that a single dose of 60% ethanol gavage about 4.7 g/kg significantly increased the serum levels of ALT and AST, exhibited obvious microvesicular steatosis, and elevated the serum and hepatic TG level in mice [46]. Collectively, these data indicate that SAV exerts a protective effect against alcohol-induced liver injury.

Oxidative stress, a major cause of alcoholic liver injury, is induced by over generation of ROS under the stimulation of alcohol [49]. Excess ROS mainly induces lipid peroxidation, as indicated by the production of MDA content [50]. MDA, a major product of lipid peroxidation, has been known as a marker of oxidative stress [51]. The current study showed that SAV pretreatment significantly suppressed the levels of ROS and MDA in alcohol-treated mice livers. It is well known that ROS generation is normally counterbalanced by antioxidant defense systems [52]. The results of our study showed that alcohol exposure significantly decreases the activities of GSH-Px SOD and CAT. However, SAV remarkably reversed the depletion of these antioxidant enzymes. Taken together, the results indicate that SAV can inhibit alcohol-induced oxidative stress by suppressing ROS and lipid peroxidation and enhancing antioxidant defense systems.

Nrf2 is a critical transcription factor, which can encode detoxification enzymes and antioxidant proteins to against oxidative stress [53]. Normally, Nrf2 is inactivated in the cytoplasm by the binding of Kelch-like ECH-associated protein 1 (Keap 1). It can be activated by stimulation of antioxidants, and binds to antioxidant-response elements (AREs) to activate the expressions of downstream genes, such as HO-1, NQO1, and γ-glutamylcysteine synthetase (γ-GCS) [54]. In the present study, the results showed that SAV reversed the depletion of Nrf2 and its downstream targets NQO1, HO-1, and GCLM in alcohol-treated liver tissues. These findings were in line with our previous study in which we found that SAV up-regulated the Nrf2-mediated antioxidant pathway to protect against hydrogen peroxide (H_2_O_2_)-induced oxidative damage in vitro [26]. Therefore, our results demonstrate that SAV exhibits hepatoprotective effect against alcohol-induced oxidative stress via activation of Nrf2 signal pathway.

Alcohol-induced translocation of endotoxin (LPS) can activate Kupffer cells, which are recognized as a crucial mediator of liver inflammation [55]. It has been reported that elevation of LPS was found in patients with alcoholic liver disease (ALD) [56]. Our results showed that LPS was significantly increased in alcohol-treated mice. However, this increase was remarkably reversed by SAV pretreatment. Furthermore, pro-inflammatory enzymes iNOS and COX-2 play important roles in LPS-induced liver damage, and the activation of iNOS can induce the production of high levels of NO [57,58]. Venkatraman et al. found that the induction of iNOS is a major contributor in the pathogenesis of alcoholic liver injury by establishing a model of iNOS-knockout mice [59]. The current findings showed that SAV significantly suppressed the increase of COX-2, iNOS and NO in the alcohol-treated group. It was reported that the activation of COX-2 and iNOS can promote the secretion of pro-inflammatory cytokines such as IL-1β and tumor necrosis factor (TNF-α), and reduce the expressions of anti-inflammatory cytokines such as IL-10 and interleukin-4 (IL-4) in Kupffer cells [60]. Our results showed that the elevation of IL-1β and the reduction of IL-10 were significantly restored by SAV pretreatment (2.500 mL/kg b.w.). Collectively, these results indicate that SAV can ameliorate LPS-induced liver inflammation in alcohol-exposure mice.

It has been reported that the TLR-induced signal pathway is a major pathway contributing to ethanol-induced hepatic inflammation [61]. The activation of TLR4 combined with MyD88 causes the activation of NF-κB, and subsequently induces the production of pro-inflammatory cytokines [36]. In the present study, our results confirmed that alcohol exposure significantly up-regulated the expressions of TLR4 and its downstream MyD88, p-IκB α and p-NF-κB p65. As we expected, the up-regulation of these proteins was remarkably inhibited by SAV administration. Taken together, these results indicate that SAV can inhibit alcohol-induced liver inflammation via inhibiting the TLR4-induced signal pathway in mice.

## 5. Conclusions

In conclusion, this study revealed that SAV effectively protected against alcohol-induced liver injury in mice. The protective effects were associated with reduction of alcohol-induced oxidative stress via inhibiting oxidant production and up-regulating Nrf2-mediated antioxidant response. Additionally, SAV also prevented alcohol-induced hepatic inflammation via suppressing TLR4-induced signal pathway. These findings imply that SAV is a functional food for liver protection, which would be useful for encouraging the future application and development of new health-care food.

## Figures and Tables

**Table 1:** Hepatic levels of ROS and MDA and antioxidant enzymes in mice.

Group	ROS (U/mg prot)	MDA (nmol/mg prot)	GSH-Px (U/mg prot)	SOD (U/mg prot)	CAT (U/mg prot)
Control	60.59 ± 3.40	2.25 ± 0.28	149.67 ± 14.05	371.56 ± 35.86	104.52 ± 24.99
Alcohol	99.87 ± 10.07 **	4.52 ± 0.62 **	82.27 ± 9.19 ***	260.00 ± 9.61 **	65.27 ± 18.86 ***
SAV (0.625 mL/kg)	85.50 ± 7.72	3.00 ± 0.60	105.47 ± 14.18	264.51 ± 13.43	86.99 ± 26.77
SAV (1.250 mL/kg)	70.71 ± 4.86 ##	2.64 ± 0.32 #	114.66 ± 5.64 #	299.86 ± 16.00 #	96.46 ± 33.57 #
SAV (2.500 mL/kg)	57.21 ± 3.47 ##	2.31 ± 0.34 #	126.64 ± 10.41 ##	310.54 ± 17.36 ##	113.54 ± 33.50 ##
AA (2.500 mL/kg)	90.61 ± 2.31	3.47 ± 0.52	103.84 ± 7.95	252.10 ± 8.40	68.58 ± 19.00

All data are expressed as mean ± SEM (*n* = 5–7). ** *p* < 0.01, *** *p* < 0.001 versus control group, # *p* < 0.05, ## *p* < 0.01 versus ethanol group.

## Supplementary Materials

The following are available online at http://www.mdpi.com/2072-6643/10/7/805/s1, Figure S1: Effects of single-dose and 14-day SAV in alcohol-treated mice.
