# Supplementary material for: Shanxi Aged Vinegar Protects against Alcohol-Induced Liver Injury via Activating Nrf2-Mediated Antioxidant and Inhibiting TLR4-Induced Inflammatory Response
Source: Nutrients. 2018 Jun 22;10(7):805. doi: 10.3390/nu10070805 (PMC6073858; doi:10.3390/nu10070805)
Supplement: Supplementary file 1 [file nutrients-10-00805-s001.pdf]

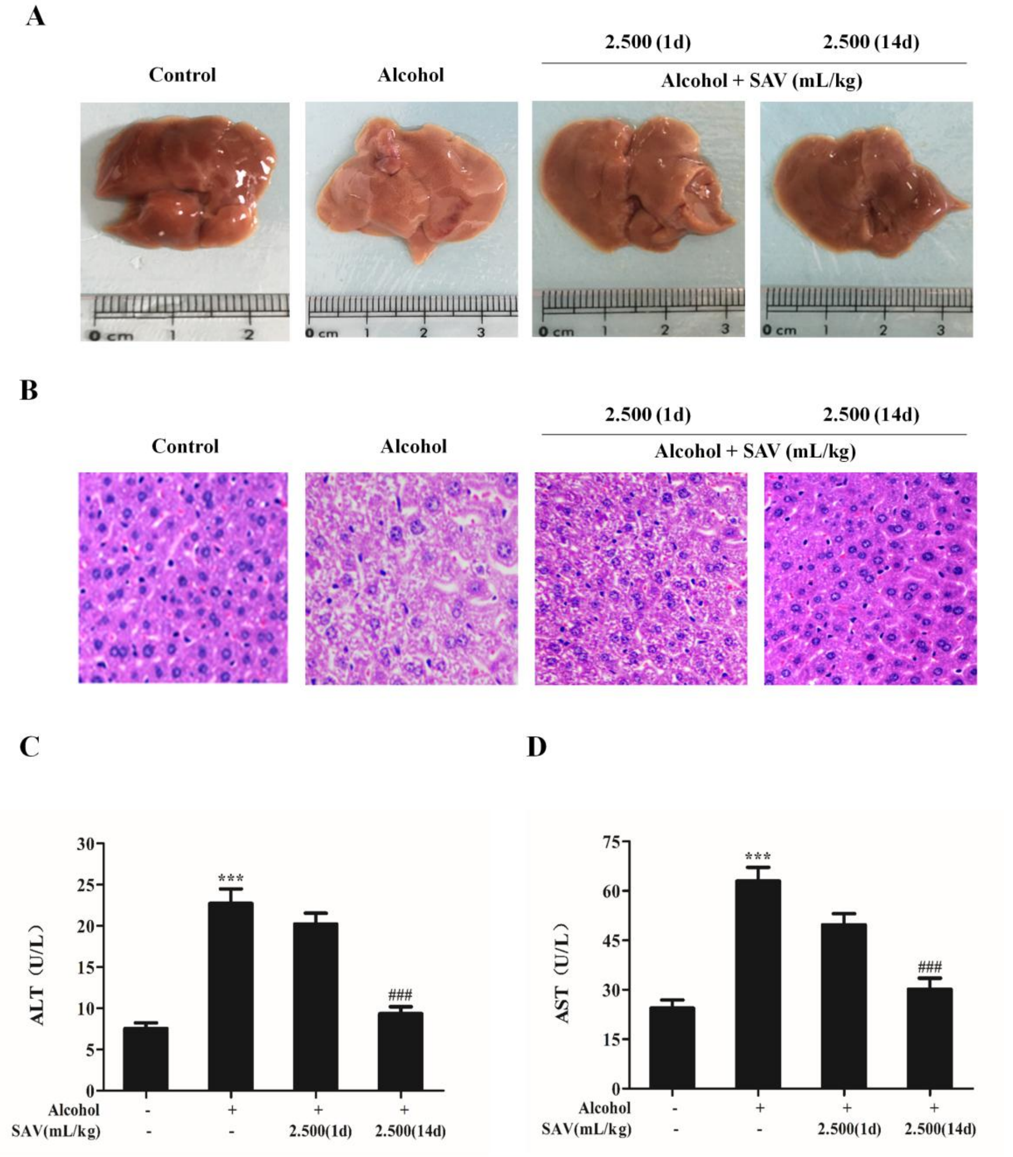

Supplementary material 1. Effects of single-dose and 14-day SAV in alcohol-treated mice. (A) Gross examination of mice livers. (B) Histological examination of liver sections stained with H&E.（200× magnification） Serum levels of ALT (C) and AST (D) were measured with microplate reader. All data are expressed as mean ± SEM (n = 5-7). $^{***}P < 0.001$ versus control group, $^{\#\#\#}P<0.001$ versus ethanol group.
